# Supplementary material for: Unveiling the Bioactive Potential of the Invasive Jellyfish Phyllorhiza punctata Through Integrative Transcriptomic and Proteomic Analyses
Source: Biomolecules. 2025 Aug 4;15(8):1121. doi: 10.3390/biom15081121 (PMC12383608; doi:10.3390/biom15081121)
Supplement: Supplementary file 1 [file biomolecules-15-01121-s001.zip › Suplementary Material/FileS3/PEPFOLD4/XP_065058878_15030/clusters.html]

 PEP-FOLD clusters


Download archive of all models  here   

|  |  |  |
| --- | --- | --- |
| # | file | sOPEP |
| # cluster 1 - Size: 10 |  |  |
|  | XP\_065058878\_15030-model1 | -11.0991 |
|  | XP\_065058878\_15030-model1.1 | -11.0854 |
|  | XP\_065058878\_15030-model1.2 | -10.2922 |
|  | XP\_065058878\_15030-model1.3 | -10.2008 |
|  | XP\_065058878\_15030-model1.4 | -10.1897 |
|  | XP\_065058878\_15030-model1.5 | -10.1608 |
|  | XP\_065058878\_15030-model1.6 | -10.0929 |
|  | XP\_065058878\_15030-model1.7 | -10.0886 |
|  | XP\_065058878\_15030-model1.8 | -9.7328 |
|  | XP\_065058878\_15030-model1.9 | -9.17018 |
| # cluster 2 - Size: 7 |  |  |
|  | XP\_065058878\_15030-model2 | -10.8271 |
|  | XP\_065058878\_15030-model2.1 | -10.4398 |
|  | XP\_065058878\_15030-model2.2 | -10.1101 |
|  | XP\_065058878\_15030-model2.3 | -9.94259 |
|  | XP\_065058878\_15030-model2.4 | -9.93385 |
|  | XP\_065058878\_15030-model2.5 | -9.89573 |
|  | XP\_065058878\_15030-model2.6 | -9.1114 |
| # cluster 3 - Size: 2 |  |  |
|  | XP\_065058878\_15030-model3 | -10.7706 |
|  | XP\_065058878\_15030-model3.1 | -10.6706 |
| # cluster 4 - Size: 5 |  |  |
|  | XP\_065058878\_15030-model4 | -10.6428 |
|  | XP\_065058878\_15030-model4.1 | -10.2524 |
|  | XP\_065058878\_15030-model4.2 | -10.2173 |
|  | XP\_065058878\_15030-model4.3 | -9.72914 |
|  | XP\_065058878\_15030-model4.4 | -9.64636 |
| # cluster 5 - Size: 3 |  |  |
|  | XP\_065058878\_15030-model5 | -10.6171 |
|  | XP\_065058878\_15030-model5.1 | -10.5555 |
|  | XP\_065058878\_15030-model5.2 | -9.9071 |
| # cluster 6 - Size: 8 |  |  |
|  | XP\_065058878\_15030-model6 | -10.5453 |
|  | XP\_065058878\_15030-model6.1 | -10.3069 |
|  | XP\_065058878\_15030-model6.2 | -10.2601 |
|  | XP\_065058878\_15030-model6.3 | -10.0952 |
|  | XP\_065058878\_15030-model6.4 | -10.0474 |
|  | XP\_065058878\_15030-model6.5 | -10.0197 |
|  | XP\_065058878\_15030-model6.6 | -9.70709 |
|  | XP\_065058878\_15030-model6.7 | -9.65437 |
| # cluster 7 - Size: 9 |  |  |
|  | XP\_065058878\_15030-model7 | -10.3548 |
|  | XP\_065058878\_15030-model7.1 | -10.2063 |
|  | XP\_065058878\_15030-model7.2 | -10.1931 |
|  | XP\_065058878\_15030-model7.3 | -10.0662 |
|  | XP\_065058878\_15030-model7.4 | -10.0197 |
|  | XP\_065058878\_15030-model7.5 | -9.89887 |
|  | XP\_065058878\_15030-model7.6 | -9.86197 |
|  | XP\_065058878\_15030-model7.7 | -9.5401 |
|  | XP\_065058878\_15030-model7.8 | -9.20171 |
| # cluster 8 - Size: 7 |  |  |
|  | XP\_065058878\_15030-model8 | -10.3025 |
|  | XP\_065058878\_15030-model8.1 | -9.79819 |
|  | XP\_065058878\_15030-model8.2 | -9.68524 |
|  | XP\_065058878\_15030-model8.3 | -9.28489 |
|  | XP\_065058878\_15030-model8.4 | -9.23171 |
|  | XP\_065058878\_15030-model8.5 | -9.11826 |
|  | XP\_065058878\_15030-model8.6 | -8.7707 |
| # cluster 9 - Size: 1 |  |  |
|  | XP\_065058878\_15030-model9 | -10.1197 |
| # cluster 10 - Size: 1 |  |  |
|  | XP\_065058878\_15030-model10 | -10.1059 |
| # cluster 11 - Size: 2 |  |  |
|  | XP\_065058878\_15030-model11 | -10.0828 |
|  | XP\_065058878\_15030-model11.1 | -9.15541 |
| # cluster 12 - Size: 1 |  |  |
|  | XP\_065058878\_15030-model12 | -9.91926 |
| # cluster 13 - Size: 4 |  |  |
|  | XP\_065058878\_15030-model13 | -9.76281 |
|  | XP\_065058878\_15030-model13.1 | -9.65834 |
|  | XP\_065058878\_15030-model13.2 | -9.26576 |
|  | XP\_065058878\_15030-model13.3 | -8.95713 |
| # cluster 14 - Size: 2 |  |  |
|  | XP\_065058878\_15030-model14 | -9.66902 |
|  | XP\_065058878\_15030-model14.1 | -8.83221 |
| # cluster 15 - Size: 3 |  |  |
|  | XP\_065058878\_15030-model15 | -9.45692 |
|  | XP\_065058878\_15030-model15.1 | -8.32112 |
|  | XP\_065058878\_15030-model15.2 | -7.90576 |
| # cluster 16 - Size: 3 |  |  |
|  | XP\_065058878\_15030-model16 | -8.94904 |
|  | XP\_065058878\_15030-model16.1 | -8.68074 |
|  | XP\_065058878\_15030-model16.2 | -8.67975 |
| # cluster 17 - Size: 1 |  |  |
|  | XP\_065058878\_15030-model17 | -8.69057 |
| # cluster 18 - Size: 1 |  |  |
|  | XP\_065058878\_15030-model18 | -7.95913 |
| # cluster 19 - Size: 1 |  |  |
|  | XP\_065058878\_15030-model19 | -7.21453 |
| # cluster 20 - Size: 1 |  |  |
|  | XP\_065058878\_15030-model20 | -6.70271 |
| # cluster 21 - Size: 1 |  |  |
|  | XP\_065058878\_15030-model21 | -6.67369 |
| # cluster 22 - Size: 2 |  |  |
|  | XP\_065058878\_15030-model22 | -6.6527 |
|  | XP\_065058878\_15030-model22.1 | -6.23944 |
| # cluster 23 - Size: 1 |  |  |
|  | XP\_065058878\_15030-model23 | -6.5077 |
| # cluster 24 - Size: 9 |  |  |
|  | XP\_065058878\_15030-model24 | -6.34968 |
|  | XP\_065058878\_15030-model24.1 | -6.22746 |
|  | XP\_065058878\_15030-model24.2 | -6.18517 |
|  | XP\_065058878\_15030-model24.3 | -6.16559 |
|  | XP\_065058878\_15030-model24.4 | -6.03378 |
|  | XP\_065058878\_15030-model24.5 | -6.02909 |
|  | XP\_065058878\_15030-model24.6 | -5.99437 |
|  | XP\_065058878\_15030-model24.7 | -5.85834 |
|  | XP\_065058878\_15030-model24.8 | -5.76324 |
| # cluster 25 - Size: 1 |  |  |
|  | XP\_065058878\_15030-model25 | -6.32381 |
| # cluster 26 - Size: 4 |  |  |
|  | XP\_065058878\_15030-model26 | -6.28723 |
|  | XP\_065058878\_15030-model26.1 | -5.99819 |
|  | XP\_065058878\_15030-model26.2 | -5.96123 |
|  | XP\_065058878\_15030-model26.3 | -5.60042 |
| # cluster 27 - Size: 3 |  |  |
|  | XP\_065058878\_15030-model27 | -6.08384 |
|  | XP\_065058878\_15030-model27.1 | -5.81594 |
|  | XP\_065058878\_15030-model27.2 | -5.50625 |
| # cluster 28 - Size: 1 |  |  |
|  | XP\_065058878\_15030-model28 | -5.88187 |
| # cluster 29 - Size: 1 |  |  |
|  | XP\_065058878\_15030-model29 | -5.85663 |
| # cluster 30 - Size: 2 |  |  |
|  | XP\_065058878\_15030-model30 | -5.83847 |
|  | XP\_065058878\_15030-model30.1 | -5.82719 |
| # cluster 31 - Size: 1 |  |  |
|  | XP\_065058878\_15030-model31 | -5.76694 |
| # cluster 32 - Size: 1 |  |  |
|  | XP\_065058878\_15030-model32 | -5.74534 |
| # cluster 33 - Size: 1 |  |  |
|  | XP\_065058878\_15030-model33 | -5.51696 |
|  |  |  |
